# Supplementary material for: Multistage Nanocarrier Based on an Oil Core–Graphene Oxide Shell
Source: Pharmaceutics. 2024 Jun 18;16(6):827. doi: 10.3390/pharmaceutics16060827 (PMC11207637; doi:10.3390/pharmaceutics16060827)
Supplement: Supplementary file 1 [file pharmaceutics-16-00827-s001.zip › pharmaceutics-2988545-supplementary.pdf]

# Supporting Information

# Multistage nanocarrier based on oil core - graphene oxide shell

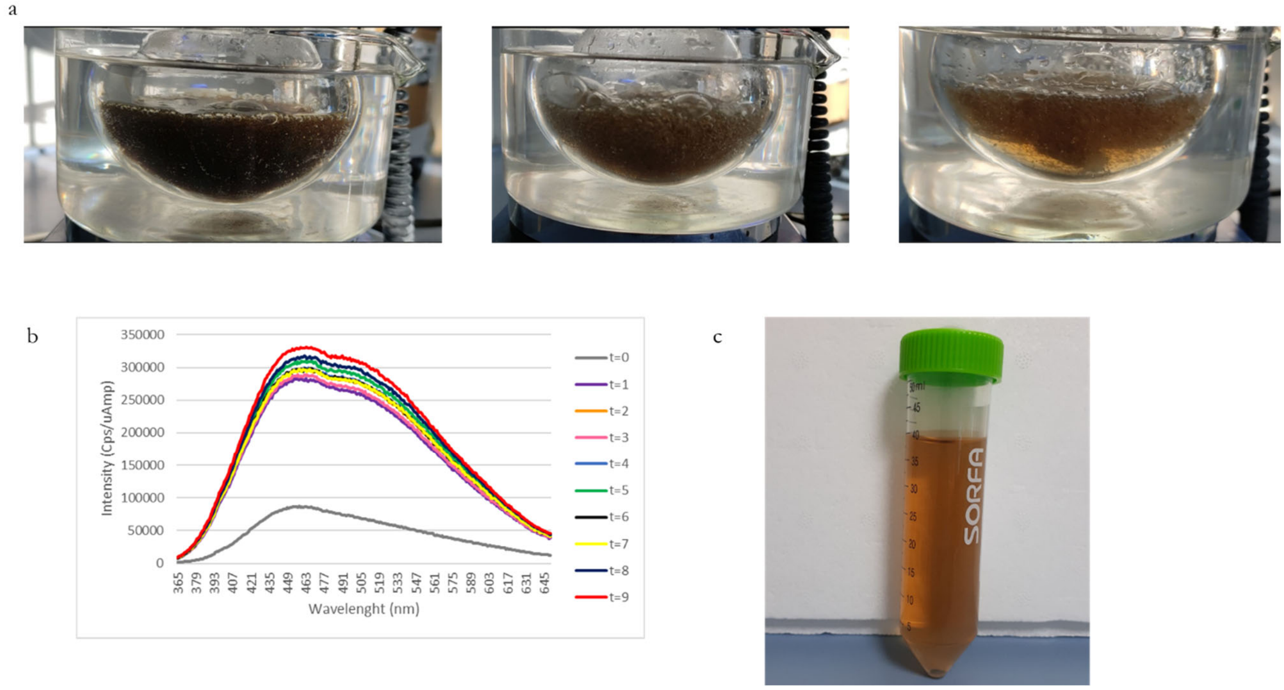


*Figure S1* *(a) Frame of the proceeding of the formation reaction of GOQDs-*$NH_2$*, (b) Temporal evolution of PL emission during preparation of GOQDs-*$NH_2$*;(c) GOQDs-*$NH_2$ *obtained after purification.*

The fluorescence intensity measurements (PL intensity) were used to monitor the progress of the GOQDs-$NH_2$ formation reaction. As shown in Fig. S1 PL appears ca. after 1 h of reaction and it reaches its maximum value after 8 h of heating at 80°C, after which the PL intensity decreases.

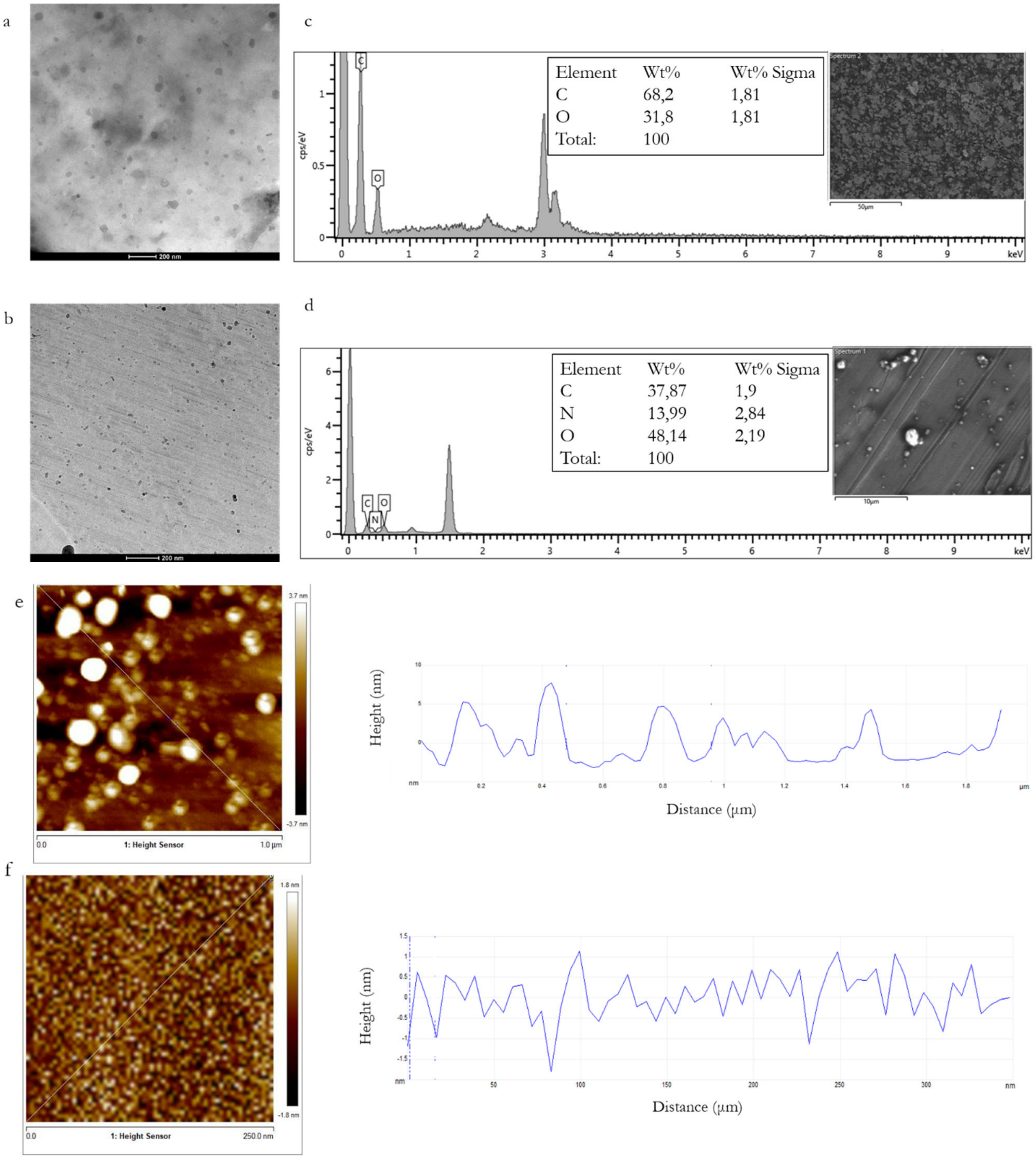


*Figure S2* *(a)TEM image $GO_{Hummer}$ (b), Cryo-TEM image GOQDs-$NH_2$ (c) EDX spectra of $GO_{Hummer}$;(d) EDX spectra of GOQDs-$NH_2$;(e) AFM Images (left) and height profile of the AFM images (right) of $GO_{Hummer}$;(f) AFM Images (left) and height profile of the AFM images (right) of GOQDs-$NH_2$.*

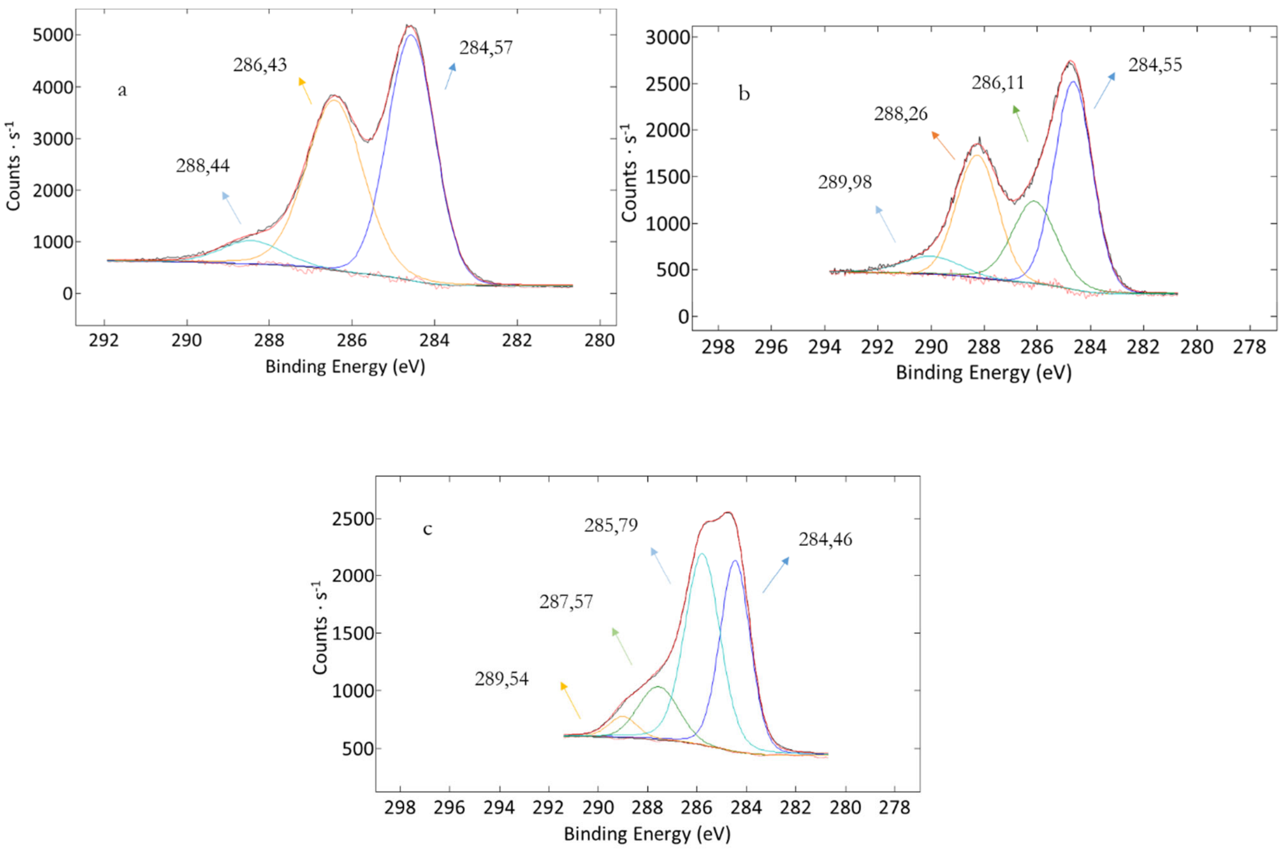


*Figure S3 De-convoluted C1s core level XPS spectra for a) $GO_{Hummer}$; b) GOQDs-$NH_2$; c) GOQDs-HA.*

| C1s area [%] | | | | |
|---|---|---|---|---|
| Sample | C-C | C-O | C-O-C | O-C=O |
| $GO_{Hummer}$ | 49.92 | / | 43.45 | 6.63 |
| GOQDs-$NH_2$ | 44.20 | 21.86 | 27.77 | 6.18 |
| GOQDs-HA | 37.67 | 45.32 | 12.95 | 4.06 |

*Table S1 Binding Energy Values in C1s level from XPS analysis*

The C1s spectrum of $GO_{Hummer}$ (Fig.S3a) showed the characteristic peak of the carbonaceous skeleton at 284.57 eV and the characteristic peaks of the oxygenated functional groups such as ether and epoxy (286.43 eV) and carbonyl/carboxyl (288.44 eV) groups. In the sample treated with the oxidative cutting (Fig. S3b) four types of carbon atoms could be detected in the C1s high resolution spectrum: graphitic C-C and C=C (284,55 eV) C-O functions (286,11 eV) and C=O and COOH groups (288,55 eV and 289.98 eV). However, the latter two functions may be present in GOQDs due to the weak energy difference between C-C or C=C and C-N or C=N functions. Similar peaks were observed in GOQDs-HA (FigS3c).

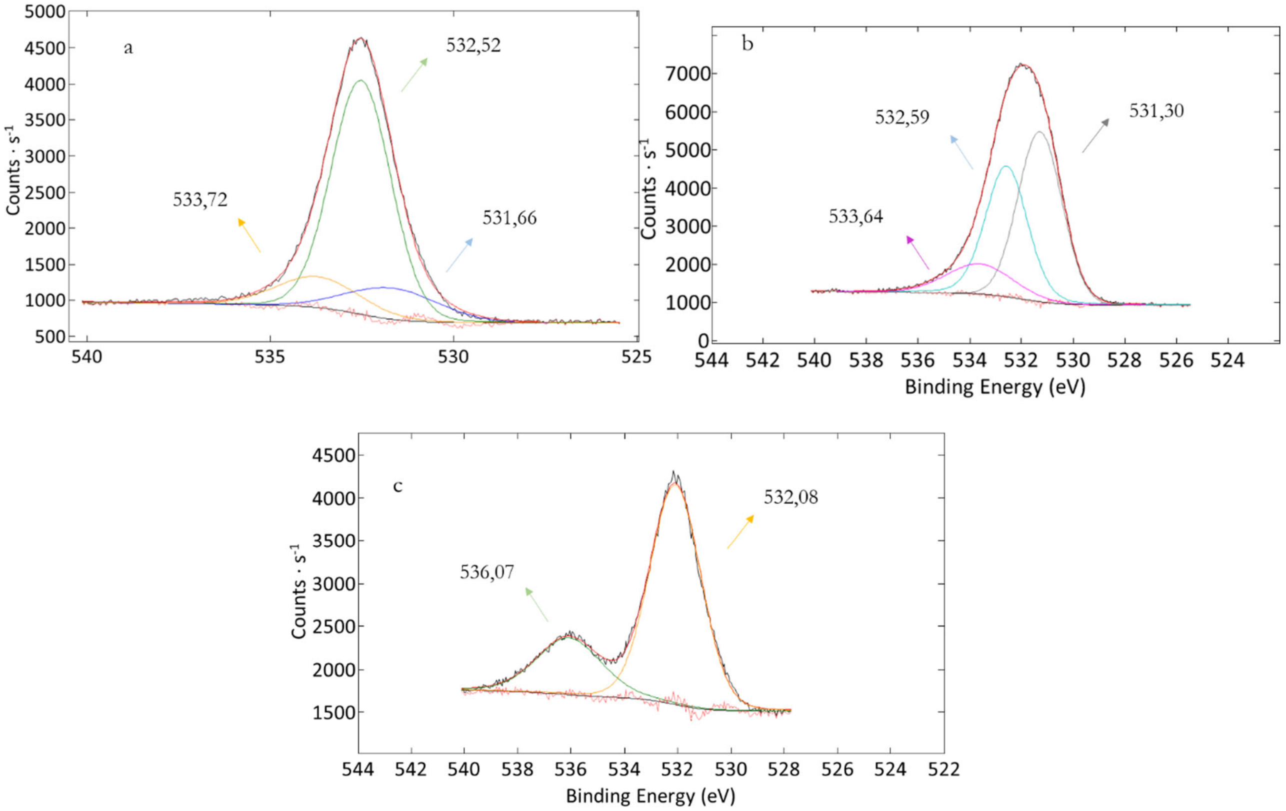


*Figure S4* *De-convoluted O1s core level XPS spectra for a) $GO_{Hummer}$, b) GOQDs-$NH_2$, c) GOQDs-HA.*

| Sample | C-O | O-C=O | O=C |
|---|---|---|---|
| $GO_{Hummer}$ | 71.96 | 13.76 | 14.28 |
| GOQDs-$NH_2$ | 48.18 | 13.73 | 38.09 |
| GOQDs-HA | 73.98 | / | 26.02 |

*Table S2* *Binding Energy Values in O1s level from XPS analysis.*

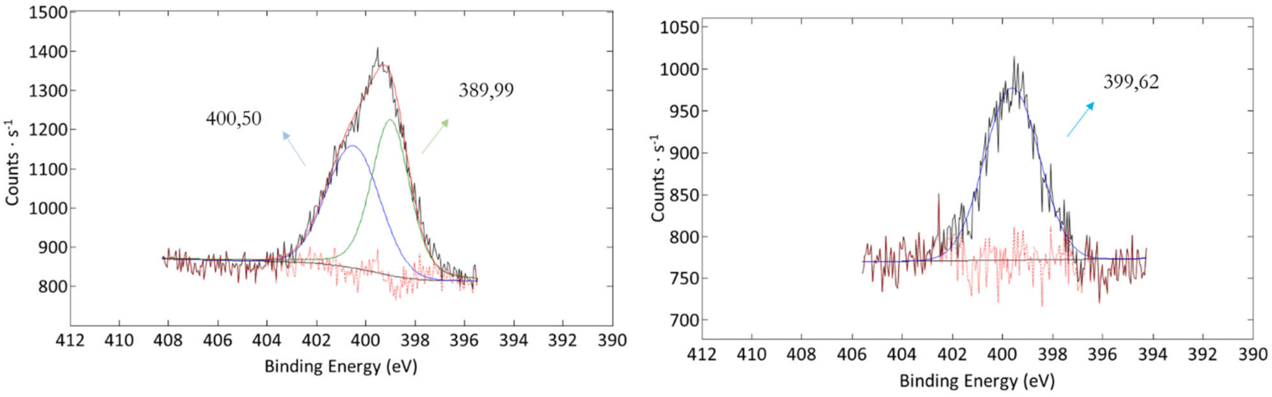


*Figure S5* *De-convoluted N1s core level XPS spectra for a) GOQDs-NH2, b) GOQDs-HA.*

| Sample | C-N/C=N | $NH_3^+$ |
|---|---|---|
| GOQDs-$NH_2$ | 51.05 | 48.95 |
| GOQDs-HA | 100 | 0 |

*Table S3 Binding Energy Values in N1s level from XPS analysis.*

a

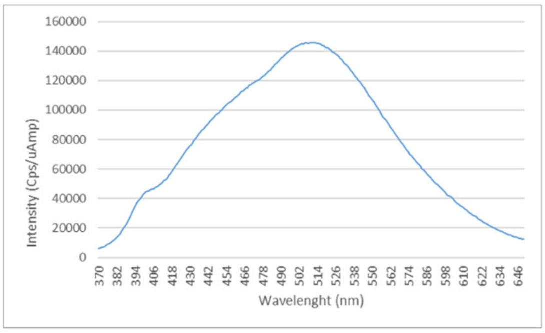


b

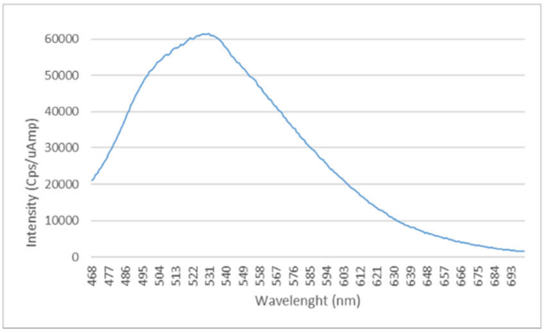


c

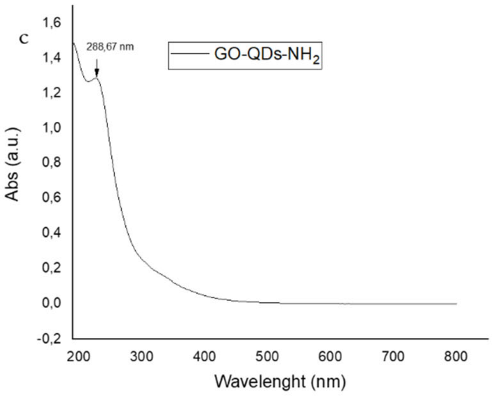


*Figure S6 a) PL emission spectrum of GOQDs-NH2, λEX 350nm, $\lambda_{EM}$ 370-650 nm; b) PL emission spectrum of GOQDs-NH2, $\lambda_{EX}$ 405nm, $\lambda_{EM}$ 648-700 nm; c) UV-VIS.*

Fig S6a,b,c show the PL emission ($\lambda_{EX}$ 350 nm 3.8b; $\lambda_{EX}$400 nm 3.8c ) and the UV-visible absorption of GOQDs-$NH_2$ after purification. The PL emission spectra of GOQDs exhibit the characteristic feature of luminescent graphene-based nanomaterials, in which the PL emission maximum is shifted to lower energy when the excitation wavelength increases. This shift originates from the optical selection of different surface defect states near the Fermi level. UV-Vis spectrum showed a characteristic peak at around 232 nm, which is attributed to π-π* transition of aromatic $sp^2$ domains.

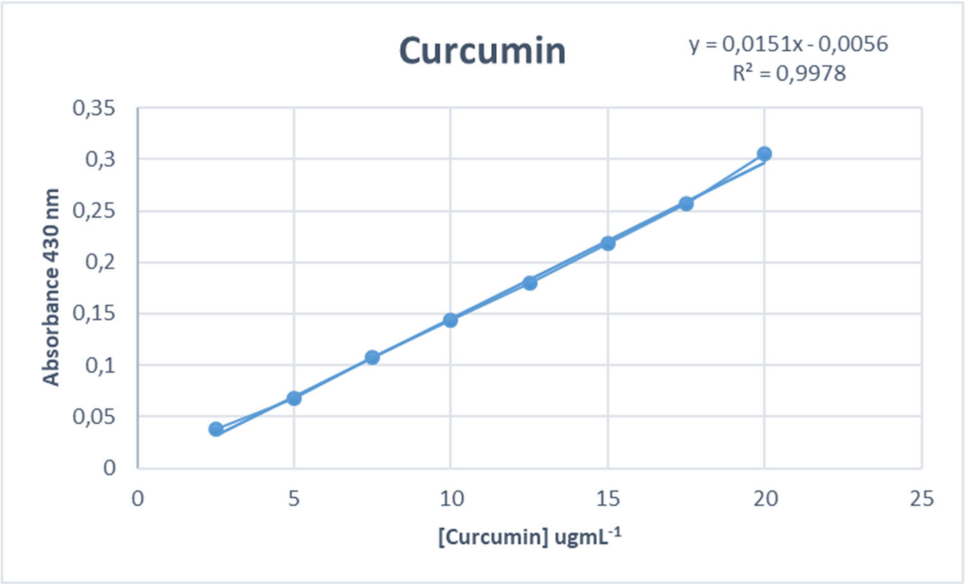


*Figure S7 Calibration Curve of Curcumin*

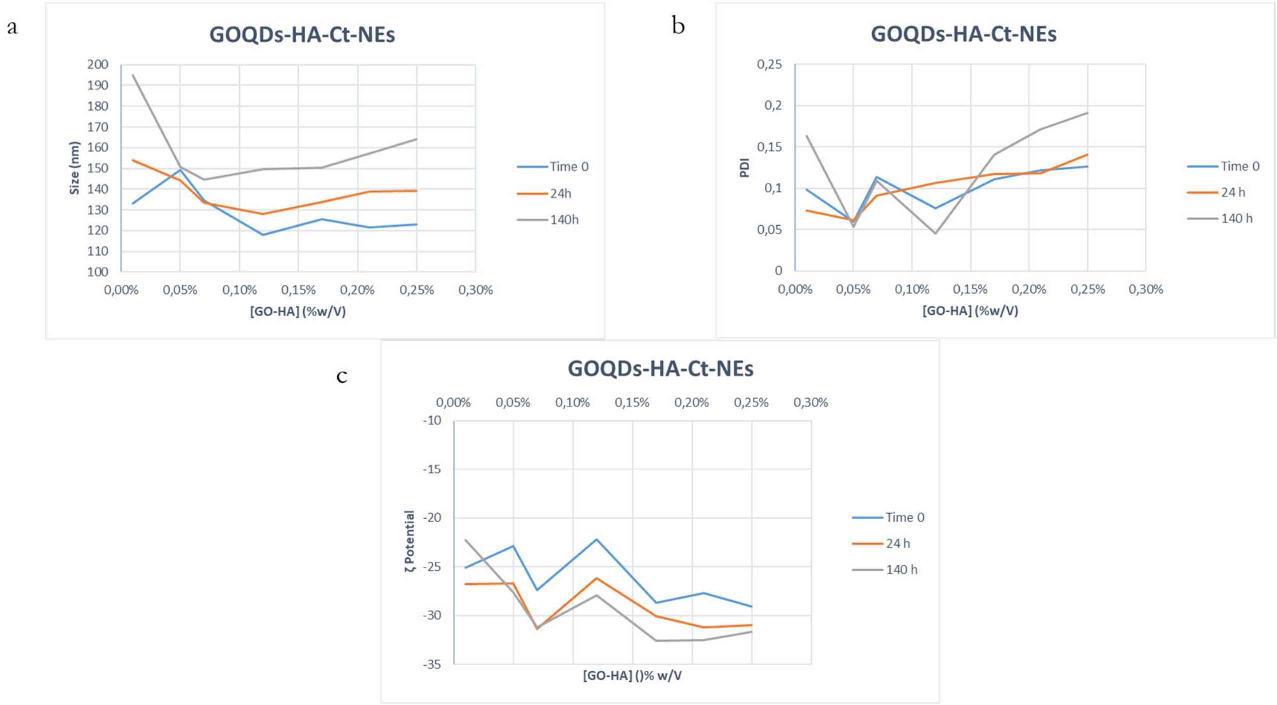


*Figure S8 Saturation Curves of GOQDs-HA-Ct-NEs.*

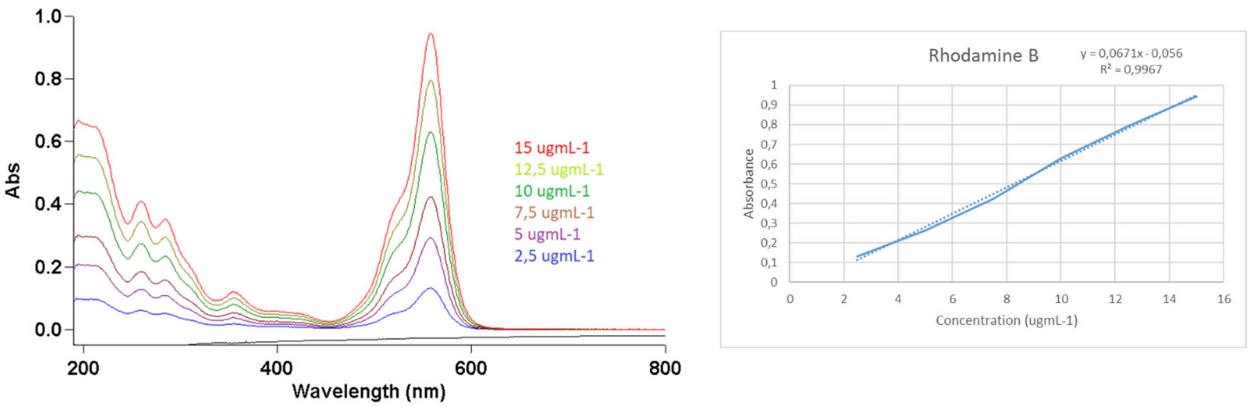


*Figure S9* *UV-Vis absorption measurements at different Rhodamine B concentrations (left) and Calibration Curve of Rhodamine B (right).*

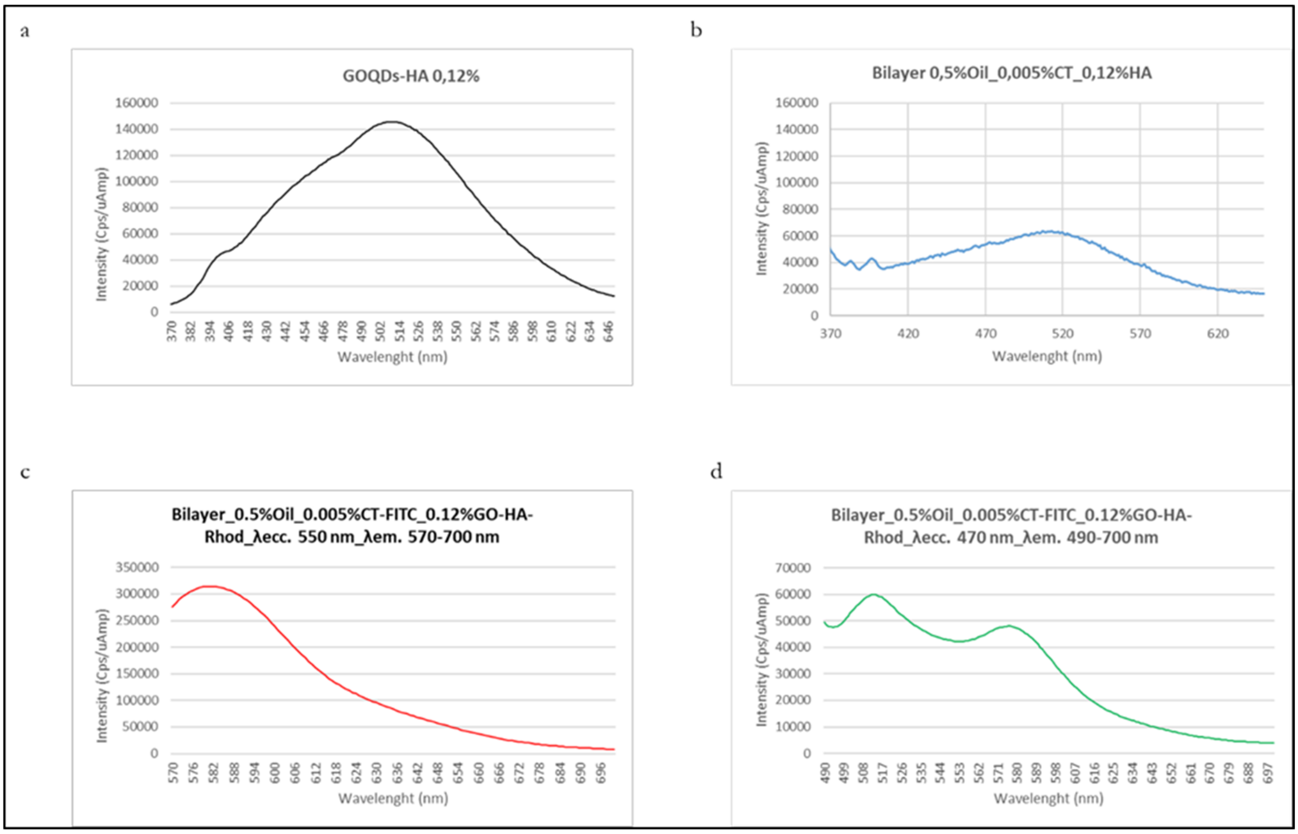


*Figure S10* *Fluorescence spectra of a) GOQDs-HA $\lambda_{EX}$ 350nm, $\lambda_{EM}$ 370-650 nm; b) Bilayer 0.5%Oil_0.05%CT_0.12%GOQDs-HA $\lambda_{EX}$ 350nm, $\lambda_{EM}$ 370-650 nm; c) Bilayer 0.5%Oil_0.05%CT-FITC_0.12%GOQDs-HA-Rhod.B; d) Bilayer 0.5%Oil_0.05%CT-FITC_0.12%GOQDs-HA-Rhod.B.*

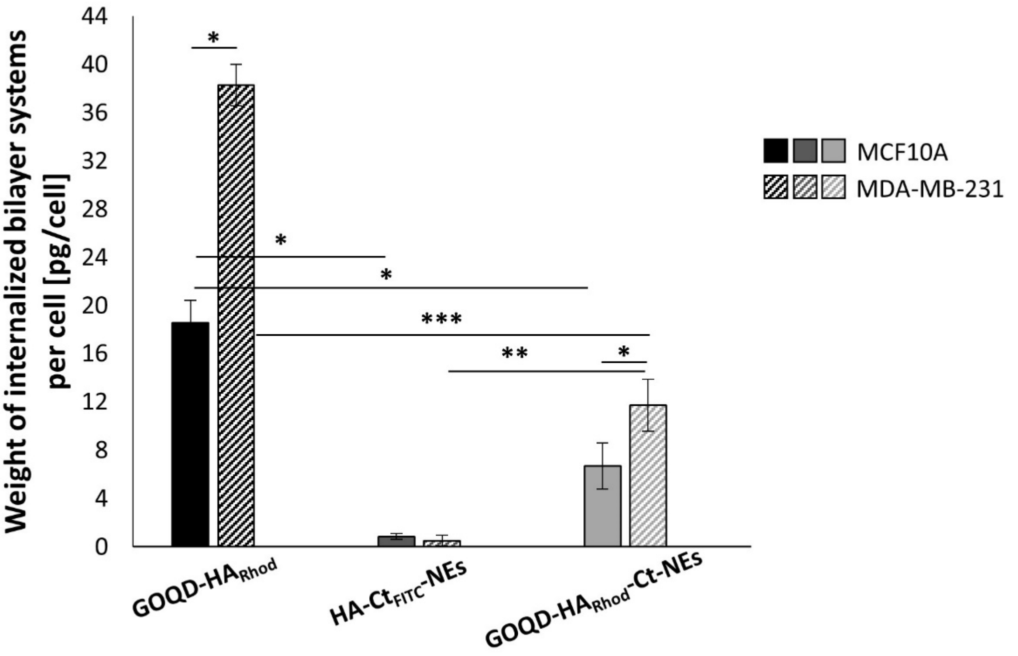


*Figure S11* *Internalization of GOQD-HARhod-Ct-NEs, HA-CtFITC-NEs and GOQDs-HA in MCF10A (solid bars) and MDA-MB-231 cells (patterned bars). *P< 0.05,**P< 0.01, ***P<0.001.*

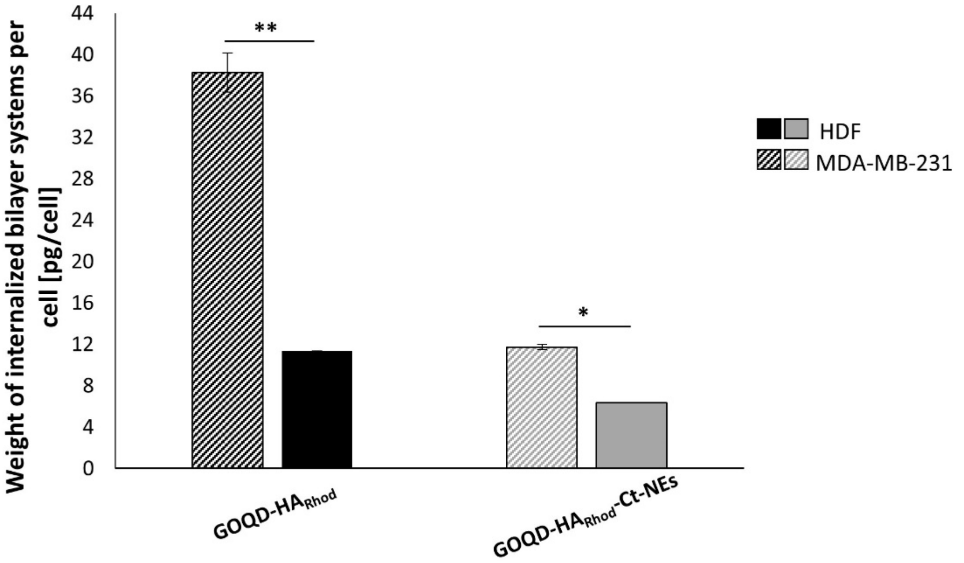


*Figure S12* *Statistical comparison among internalization data of GOQDs-HA and GOQD-HARhod-Ct-NEs in HDFs (solid bars) and MDA-MB-231 cells (patterned bars) already reported in Figures 8 and S11. *P< 0.05,**P< 0.01.*

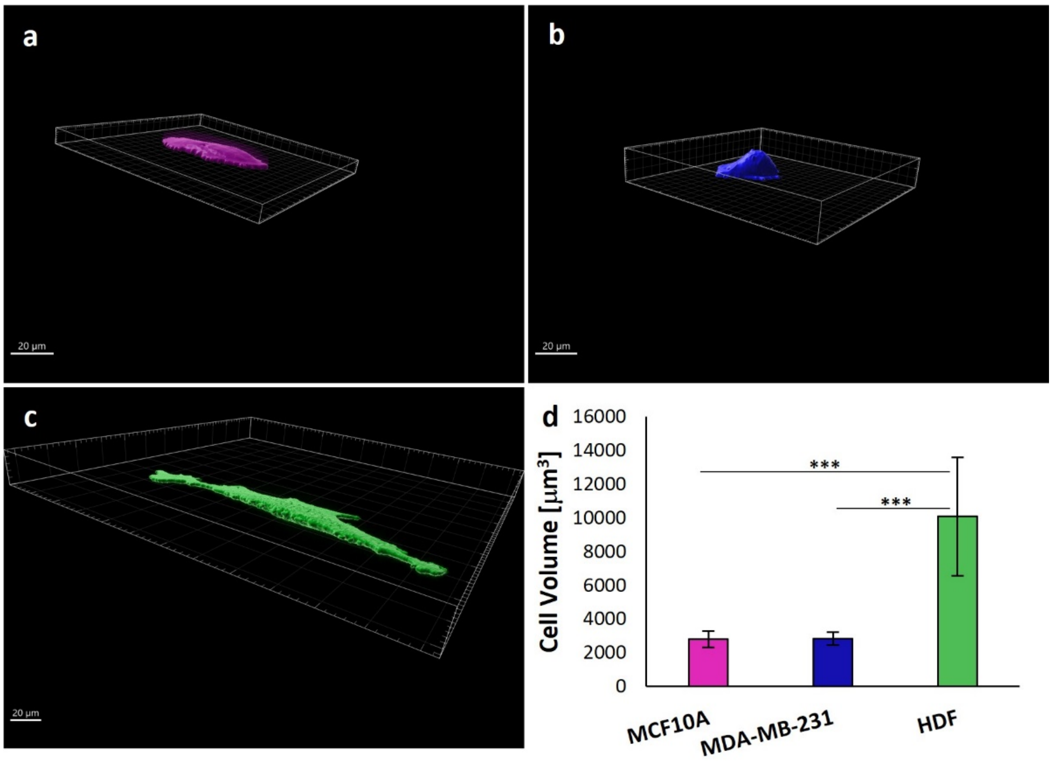


*Figure S13* *Representative images of 3D cellular reconstructions of MCF10A (a), MDA_MB-231 (b) and HDF (c). Cell volumes of MCF10A, MDA-MB-231 and HDF cells (d) reported as mean ± S.D. ***P< 0.001.*

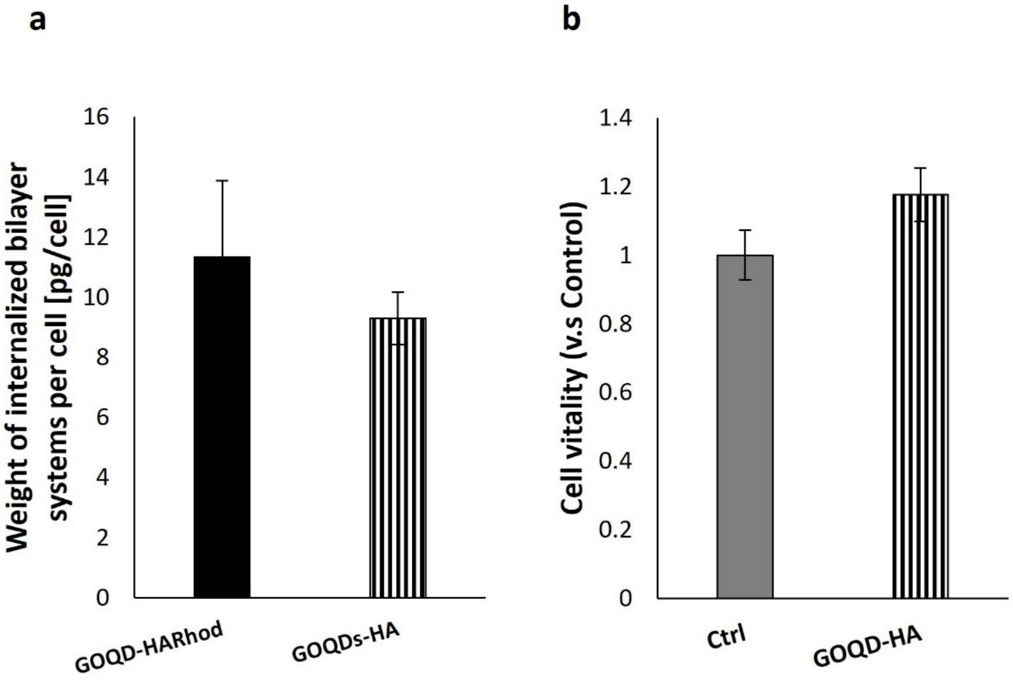


*Figure S14* *(a) Internalization of GOQDs-HA-Rhod and GOQDs-HA in HDF cells. (b) Cell viability of HDFs treated with 0.01mg/ml of GOQDs-HA. No significant differences among cell viabilities were found.*
